# Postcoital Vaginal Laceration in a Nursing Mother: A Rarely Reported Entity

**DOI:** 10.7759/cureus.47252

**Published:** 2023-10-18

**Authors:** Sudwita Sinha, Jyoti Singh, Mukta Agarwal, Indira Prasad

**Affiliations:** 1 Obstetrics and Gynaecology, All India Institute of Medical Sciences, Patna, Patna, IND

**Keywords:** parous woman, vaginal laceration, nursing mother, vaginal injury, post coital bleeding

## Abstract

Postcoital vaginal injury is an uncommon entity in parous women, although it is a commonly encountered problem in virgins. Herein, we present a case of postcoital vaginal injury after consensual intercourse in a seven-month postpartum lactating woman, leading to a 5 cm laceration between the left lateral vaginal wall and posterior vaginal fornix. There was no evidence of colporrhexis. Ultrasonography was done to rule out any intraperitoneal collection or any broad ligament hematoma. The vaginal laceration was repaired in double layers. Postoperatively, the patient had an uneventful recovery. It should be emphasized that, even though uncommon, postcoital vaginal injuries can also occur in parous women.

## Introduction

Postcoital bleeding, which is unrelated to menstruation, is spotting or bleeding that happens after sex. The percentage of menstrual women who experience postcoital bleeding varies from 0.7% to 9.0% [1]. Postcoital bleeding is typically caused by lesions on the genital surface, such as cervical polyps, cervicitis, ectropion, cervical intraepithelial lesions (CIN), or cancer [2]. Vaginal perforation is typically linked to a history of pelvic surgery, most frequently a hysterectomy. However, there are other risk factors, such as abstinence, old age, genital infections, particular coital positions, and congenital genital anomalies [3]. The presentation, diagnosis, and treatment of postcoital vaginal injuries are all poorly documented. Most of the time, these wounds are not life-threatening, but a delay in detection might cause serious complications. Embarrassment might occasionally cause anamnesis more challenging, and the diagnosis may take longer. Despite the fact that numerous methods have been established, surgical intervention is usually required to treat vaginal injuries. Postcoital vaginal injuries, although commonly seen in young virgin girls, are hardly encountered in a parous woman. Hence, we would like to emphasize the fact that, although unexpected, postcoital vaginal injuries can occur in parous women too, which may require timely diagnosis and management.

## Case presentation

**Figure 1:**
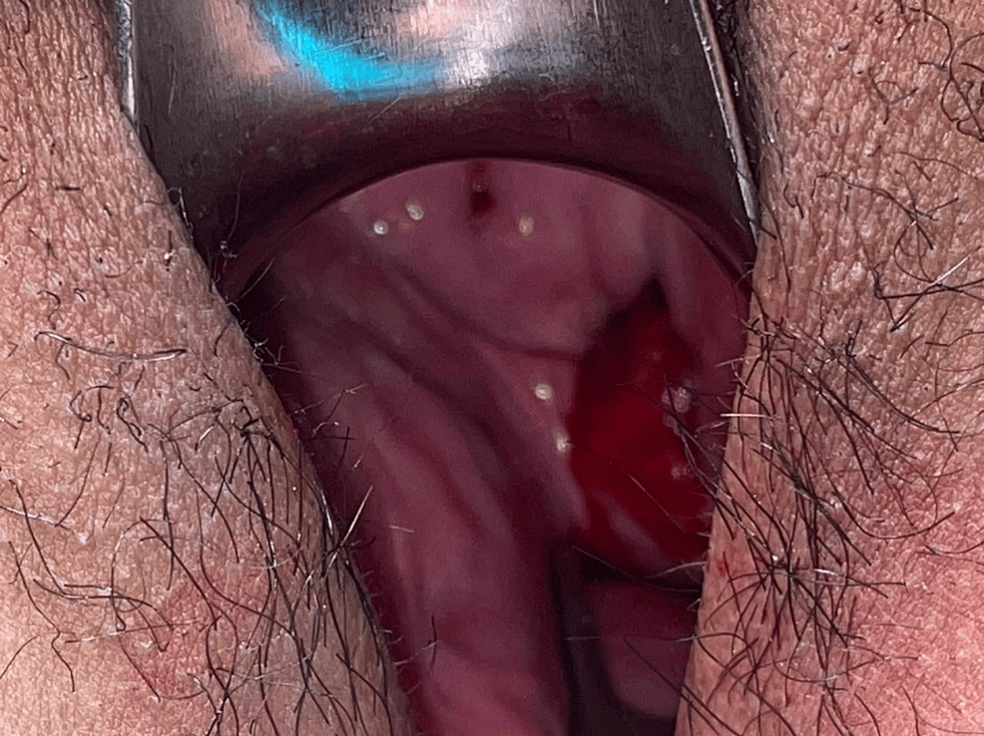
Vaginal laceration as seen on per speculum examination.

A 26-year-old parity 1, living issue 1, lactating woman presented to the emergency room two hours after having consensual vaginal intercourse with her partner of three years, with profuse vaginal bleeding and sudden onset vaginal pain. The patient was in the dorsal position during the coitus, and the spouse was on top. She denied using a foreign object, committing rape, or having forceful intercourse. She just underwent one eutocic vaginal delivery 7 months ago at our institution, and neither her obstetric injuries nor her excessive bleeding made it possible for us to conclude that she may have damaged some vaginal tissue during her delivery. Also, she had been having consensual intercourse with her partner for two months after delivery without any difficulty. She was afebrile but had tachycardia (a pulse rate of 110 beats per minute) when she presented, and her blood pressure was 120/70 mmHg. Pallor was present. An abdominal exam revealed lower quadrant palpable discomfort without rebound tenderness or rigidity of the abdominal wall. No vulval hemorrhage or laceration was seen on inspection during the perineal exam. Upon vaginal examination, a 5 cm laceration between the left lateral and posterior vaginal fornix was seen, which was bleeding. Mucosa and muscles were intact during a rectal examination. Figure 1 shows the vaginal laceration as seen on a per speculum examination.

**Figure 2:**
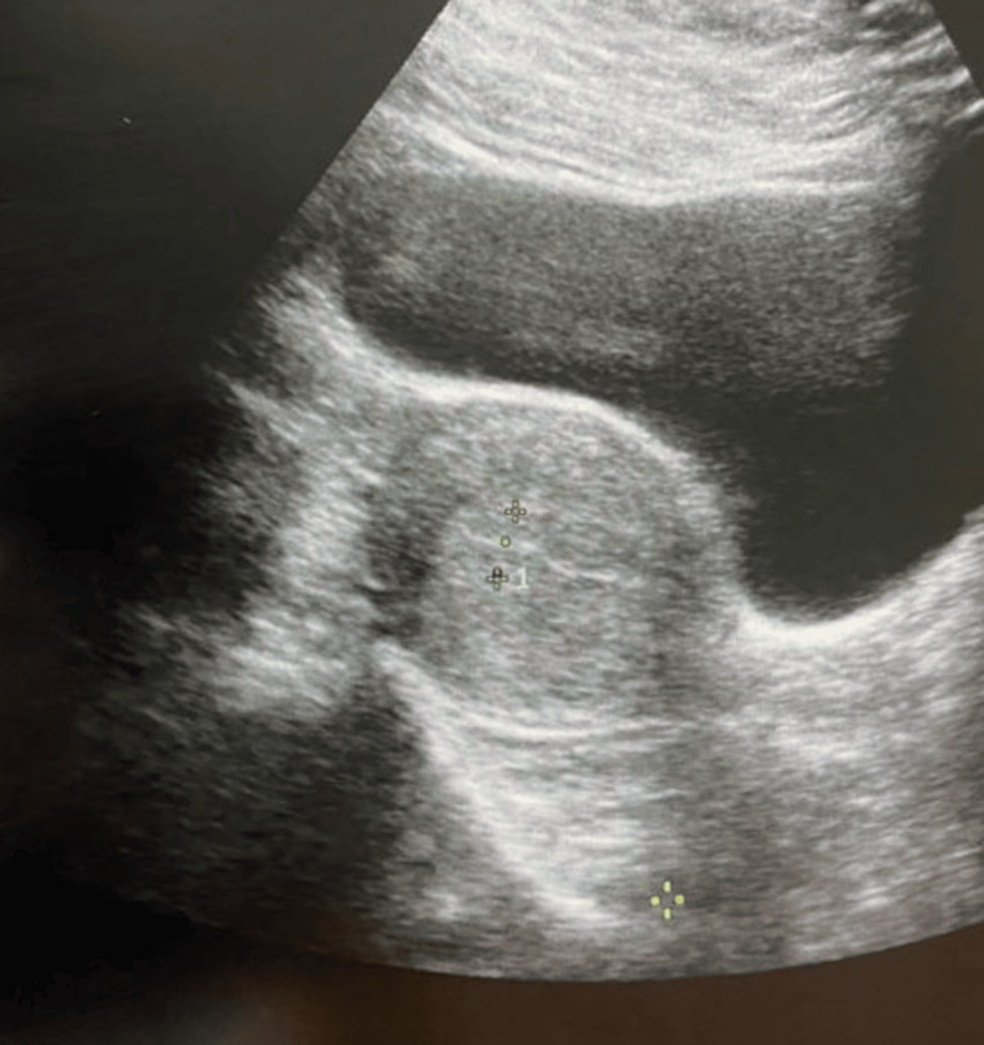
Ultrasonography of the pelvis with normal findings of uterus and no signs of intraperitoneal hemorrhage.

Her blood investigations showed a white blood cell count of 8500 per cubic mm, a platelet count of 242,000 per cubic mm, and a normal coagulation profile. However, her hemoglobin level was 7.2 g/dl. An immediate ultrasonography was performed to exclude intraperitoneal collection or broad ligament hematoma. Figure 2 shows an image of ultrasonography of the pelvis with normal findings of the uterus and no signs of intraperitoneal hemorrhage.

**Figure 3:**
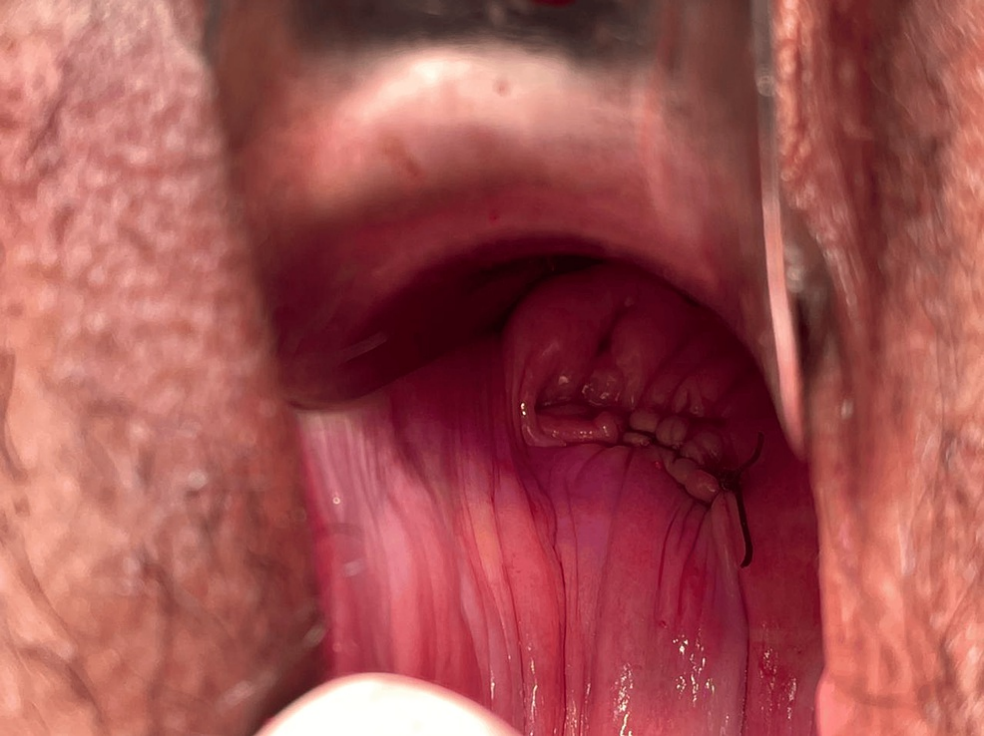
Image of the vagina after repair of the laceration.

As the patient was having significant vaginal bleeding, she was prepared for surgical repair after receiving informed consent and proper preparations. General anesthesia was administered. Surgical repair of the vaginal laceration was done with a double layer of polyglactin 910 2-0 round body suture. Figure 3 shows an image of the vagina after repair of the laceration.

Vaginal packing was done, and 2 units of packed red blood cells were transfused. The patient received parenteral antibiotics following surgery. The patient was clinically and vitally stable on her first postoperative day. Her repeat hemoglobin level after 2 units of blood transfusion was 10.2 g/dl. The vaginal pack was removed after 24 hours, after which there was no sign of active bleeding. Therefore, the patient was discharged with advice to refrain from sexual activity for six weeks to allow the operative site to heal completely. The patient was followed up in out patient clinic after six weeks. She had no complaints, and on a speculum examination, the scar site was healthy.

## Discussion

Obstetrics is the most common cause of genital female tract damage, and coitus is the second most common [4]. The vagina expands in size and becomes lubricated with transudate as a result of the sexual excitation. Intercourse that does not involve the vagina's physiological preparation is more likely to result in vaginal damage [5]. Young age, abstinence, drug and alcohol usage, infections, postmenopausal period, congenital malformations, increased uterine vascularity during pregnancy, strong sexual activity, dorsal decubitus position, vaginismus, and genital disproportion are among the risk factors [4]. Rarely, vaginal perforation might be accompanied by intra-abdominal fluids protruding with or without the hemoperitoneum. In these situations, the patient is in jeopardy of death and needs immediate medical attention. Complications including shock, peritonitis, intestinal blockages, and hollow organ perforation may occur as a result of delayed treatment [6]. In addition, a lot of patients are highly uncomfortable and may give an inaccurate or partial history. Due to this, family members or partners should not be present while the doctors perform the examination.

The posterior vaginal fornix, which is strained during coitus and appears to be more prone to injury due to a weaker layer of endopelvic fascia, is a common site of laceration [7]. The uterus' retroversion may also be a risk factor because it directs penile thrusting into the posterior fornix, which increases the risk of vaginal damage there, especially when the person is supine and the hips are hyperflexed [8]. Due to their lower-than-normal estrogen levels, caused by high prolactin levels, breastfeeding women frequently experience vaginal dryness [9]. Estrogen plays a significant role in vaginal lubrication; therefore, women who are nursing may need to use more lubricant during sex [9].

Postcoital vaginal injuries in lactating mothers are not as commonly reported as those in first-time intercourse cases. Even after an extensive review of the literature, we could hardly find a few similar cases reported in the literature, which included those reported by Aditya et al. and Nair et al. [9,10].

## Conclusions

Postcoital vaginal injuries may occur not only in virgins, postmenopausals, or forceful, unnatural sex but also in parous women, particularly nursing mothers. The location and characteristics of the lesion should be rapidly determined by a quick vaginal and rectal examination. Vaginal perforation should be ruled out and immediate surgical repair is done.
